# Supplementary material for: Probing a battery electrolyte drop with ambient pressure photoelectron spectroscopy
Source: Nat Commun. 2019 Jul 12;10:3080. doi: 10.1038/s41467-019-10803-y (PMC6626006; doi:10.1038/s41467-019-10803-y)
Supplement: Supplementary file 1 — Supplementary Information [file 41467_2019_10803_MOESM1_ESM.pdf]

Supplementary Information

Probing a battery electrolyte drop with ambient pressure photoelectron spectroscopy

Julia Maibach et al.

Supplementary Figure 1

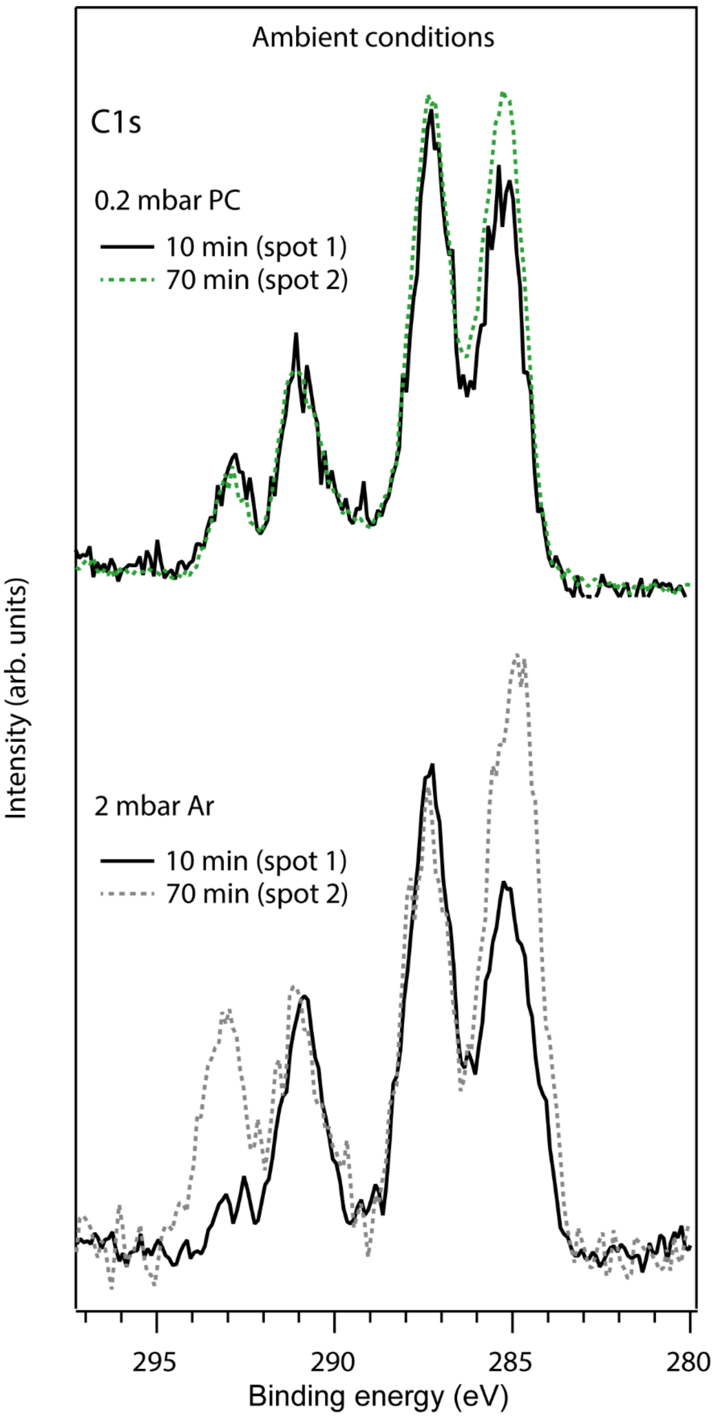

Supplementary Figure 1:

APPES measurements at different ambient conditions

C 1s spectra of Elect-Drop sample under different ambient conditions, 0.2 mbar PC or 2 mbar Ar measured after 10 and 70 minutes.

Supplementary Figure 2

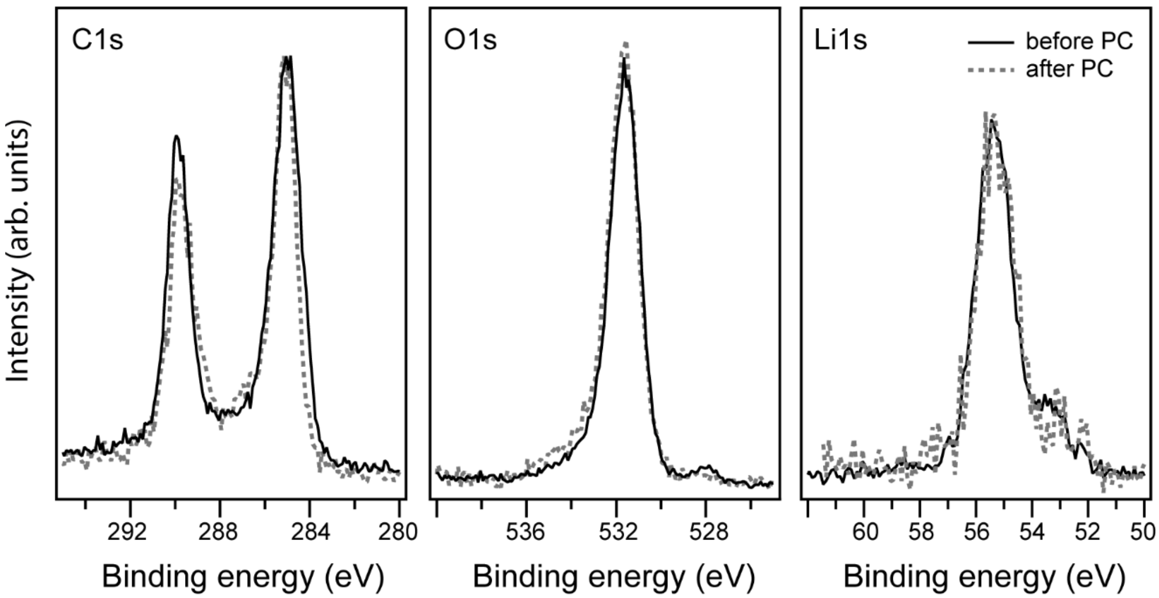

Supplementary Figure 2:

Lithium substrate stability before and after PC gas exposure

C 1s, O 1s, and Li 1s spectra of Li substrate before (solid black line) and after PC gas exposure (dotted grey line) both measured at vacuum conditions.

Supplementary Figure 3

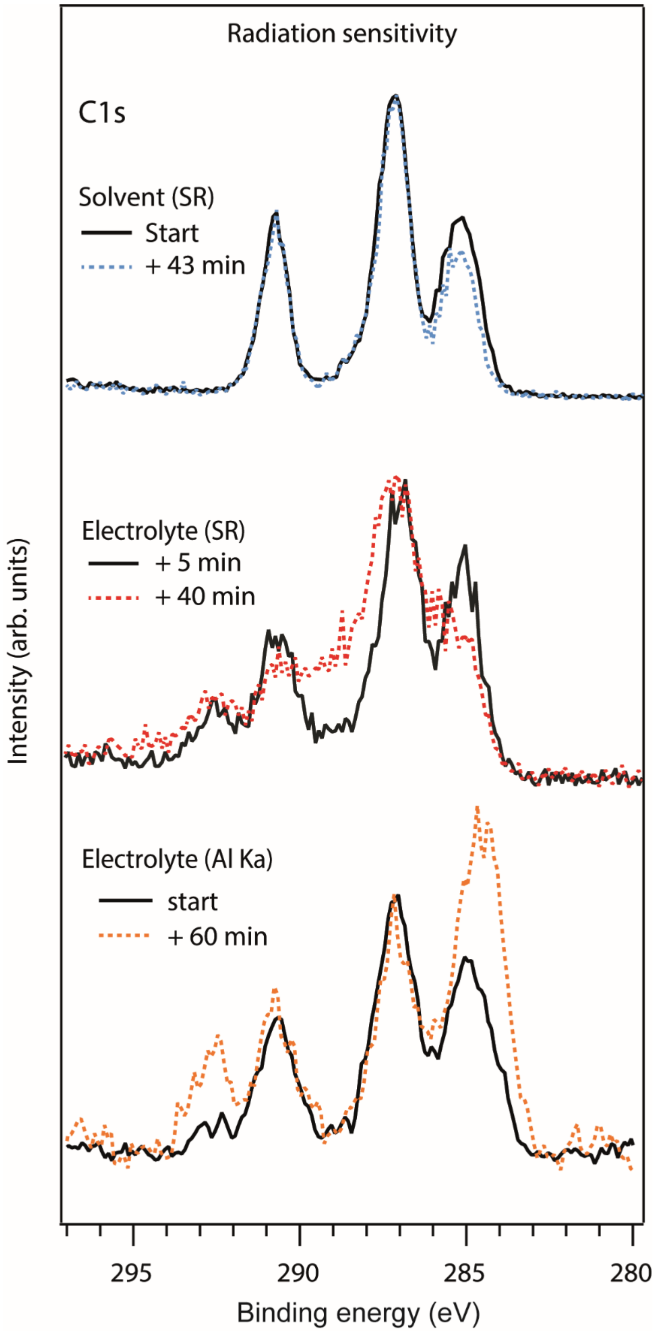

Supplementary Figure 3:

Sample stability under light exposure from different radiation sources

C 1s spectra of Solv-Drop and Elect-Drop in 0.2 mbar PC ambient conditions under continuous synchrotron radiation exposure as well as Elect-Drop in 2 mbar Ar under continuous Al Kα radiation. All samples are measured at the beginning of light exposure and after 43, 35 and 60 min, respectively. The intensities are normalized with respect to the C-O peak at 287 eV.

Supplementary Table 1

Peak areas for the evaluation of the PC to TFSI ratio in the Elect-Drop sample

Measurement spot	Area C-O (C2 PC, 286.9 eV) (cps*eV)	Area CF_3 (TFSI, 292.6 eV) (cps*eV)	PC:TFSI ratio
**I**	9477	2080	4.6
**II**	10198	2126	4.8
**III**	52740	8034	6.6
**IV**	9747	2118	4.6
**V**	52902	8811	6

Supplementary Table 2

Calculation parameters for the evaluation of the relative amounts of the different salt components in the LiTFSI electrolyte drop

Element	Peak area (cps*eV)	Cross section (800 eV from Ref. 1)	Calculated IMFP	Calculated ratio C:X	Stoichiometric ratio C:X
C (CF_3)	2080	7.70E-02	20.06	2:8.4	2:6
F ($TFSI^-$)	13656	2.92E-01	8.28		
C (CF_3)	2126	7.70E-02	20.06	2:3.8	2:4
O ($TFSI^-$)	7060	2.04E-01	13.16		
C (CF_3)	2118	7.70E-02	20.06	2:1.5	2:2
S ($TFSI^-$)	1604	6.70E-02	23.12		
C (CF_3)	8034	7.70E-02	20.06	2:1.6	2:1
N ($TFSI^-$)	9264	1.33E-01	16.85		
C (CF_3)	8811	7.70E-02	20.06	2: 12.3	2:1
Li (Li^+)	4765	5.20E-03	26.12		

Supplementary Note 1

Ambient conditions

In a previous publication, we showed that solvent evaporation occurs from an electrolyte drop in ambient conditions of N_2[1]. Therefore, we also investigated the stability of the Elect-Drop sample in ambient PC vapor environment. In Supplementary Figure 1, the spectra from the Elect-Drop samples after 10 and 70 minutes under ambient conditions of 0.2 mbar PC (835 eV excitation energy) and 2 mbar Ar (1487 eV excitation energy) are compared. Clearly, the ambient conditions of PC stabilizes the stoichiometry of the electrolyte drop well beyond the timeframe of the measurement, since the only difference between the initial and the final measurement is a slight increase in hydrocarbon species. Specifically, the salt to solvent ratio is fully preserved. However, for the ambient conditions of 2 mbar Ar, clear changes in the stoichiometry of the electrolyte are visible and the relative ratio of salt ($\mathbf{C}F_3$) to solvent (**C**-O) has changed from 1:11 to 1:2 after 70 minutes. Importantly, stabilizing the solvent was only possible in 0.2 mbar PC ambient conditions, i.e. not under Ar or N_2 ambient conditions, preventing a similar comparison for the Solv-Drop sample.

Supplementary Note 2

Electrolyte composition - Stoichiometric calculations

To determine the ratio between the solvent to salt ratio in the probed volume of the Elect-Drop sample, the integrated intensities of the C-O component for PC (C2) and the intensity of the CF_3 component for TFSI were compared for 5 different measurement positions on the same drop.

This estimation does not consider the small changes in the PC related intensities in the electrolyte (Elect-Drop sample) as compared to the solvent (Solv-Drop sample) as discussed in the main manuscript.

Since these calculations are performed for peaks of the same element in the same spectra intensity variations due to the spectrometer, analyser, sample setup and cross-sections are eliminated. Thus, the only parameter necessary for the evaluation is the peak area. This value could vary slightly depending on the peak fitting if there are several overlapping peaks. Therefore, only the C2 component of PC at 287 eV was considered and not the C3 component at 290.6 eV corresponding to the carbonate moiety in PC, since the latter might be overlapping with CF_x components. The C-O and CF_3 peaks are well separated from other contributions and therefore we believe the results to be reliable.

Supplementary Note 3

Radiation sensitivity

LiTFSI salt decomposition was observed during APPES measurements of the Elect-Drop sample. In the battery environment, LiTFSI is considered as a relatively stable salt. To follow its electrochemical stability in operando APPES experiments, it is therefore important to control radiation exposure and monitor changes if radiation damage cannot be avoided. We compared the radiation sensitivity of the Solv-Drop sample (pure PC) to the Elect-Drop sample by repeatedly measuring on the same spot. Since the carbon spectrum contains information from the solvent and salt, this region was chosen for evaluation, and the results for the Solv-Drop and the Elect-Drop samples are shown in Supplementary Figure 3 (top and middle).

From these measurements we determine, that within the time of the experiment PC is more stable versus radiation, while the electrolyte shows instability during X-ray exposure. The stability of PC is seen from the constant relative intensities of the C 1s peaks. Comparing the successive C 1s measurements of the Elect-Drop sample shows instead a relative increase of the -C-O peak with increasing measurement time. In both cases, the radiation exposure lasted roughly 40 min at a photon energy of 835 eV, i.e. at the synchrotron light source. Methodologically, it is thus vital to minimize the radiation exposure time of the electrolyte and constantly monitor the spectral changes due to radiation damage. The radiation sensitivity of the LiTFSI salt most likely stems from the fluorine atoms, which generally seem sensitive[2]. For the evaluation of the salt composition as presented in Figure 2, the measurement spot was therefore repeatedly changed to minimize radiation exposure and a C 1s line was recorded first to ensure that the electrolyte had not started to degrade.

It is notable, that this radiation sensitivity of the electrolyte is most pronounced at the synchrotron facility and thus seems to scale with the incoming photon intensity, since the repeated measurement of the C1s emission at 2 mbar Ar with Al Kα after 60 min exposure showed no additional peaks but merely the already described solvent evaporation (see Supplementary Figure 3, bottom).

Supplementary Note 4

Salt Stoichiometry in Electrolyte Drop

For the evaluation of the electrolyte composition and stoichiometry of the salt, the relative atomic ratios were calculated according to Supplementary Equation 1.

$$n_i = \frac{I_i/(\sigma_i \cdot \lambda_i)}{I_i/(\sigma_i \cdot \lambda_i) + I_{\mathrm{CF_3}}/(\sigma_{\mathrm{CF_3}} \cdot \lambda_{\mathrm{CF_3}})} \qquad (1)$$

Tabulated cross sections σ_i for the specific elements for an excitation energy of 800 eV were used[3]. The value for S2p was used without further polarization correction. Further correction factors such as transmission functions were not considered. As all spectra were recorded with the same photon energy, the different element specific photoelectrons will have different kinetic energies and thus different inelastic mean free paths (IMFP) λ_i. These were therefore calculated for photoelectrons traveling through PC based on the TTP-2M equation implemented in the NIST IMPF database. A density of 1.204 g cm^{-3} and a band gap of 9 eV for PC[4] were used as input parameters.

When evaluating the salt stoichiometry only peaks that could be identified as stemming from the intact salt were included. This means that some elements (N, Li) could be overestimated since possible degradation products would still appear at similar binding energies. For S, C and F distinguishable chemical environments can be seen, allowing us to omit these contributions for the calculations. For oxygen further difficulties occur to exactly determine the salt content since the peak is overlapping with the solvent peak.

Keeping these difficulties in mind, we acknowledge that we can have rather large variations in the calculated ratios, also due to some uncertainties in the assumptions made to estimate σ_i and λ_i[3]. Therefore, we refrain from drawing conclusions for deviations on the scale up to ~50 %, but note that the lithium content exceeds all other variations by far.

Supplementary Note 5

LiF evaluation

In order to evaluate if F^- can act as a counter-anion for the clear intensity increase in ionized lithium at the droplet surface, the relative intensity contribution from F^- (seen at a binding energy of 684.7 eV) to the Li 1s spectrum is evaluated. The intensities of the F 1s and Li 1s spectra of the Elect-Drop were firstly normalized to the corresponding CF_3 intensity, since each elemental core line was recorded individually at a fresh sample spot and the overall spectral intensity varied significantly between the different measurement spots. In the F 1s spectrum, the contributions from the $TFSI^-$ and F^- are clearly separated. Therefore, these normalized intensities are used together with the known atomic ratios of F to Li (6 to 1 in LiTFSI and 1 to 1 in LiF, respectively) to reversely calculate the expected Li 1s intensity contributions using Supplementary Equation 2.

$$I_{Li} = \frac{n_{\mathrm{Li}}}{n_{\mathrm{F}}} \cdot \frac{(\sigma_{\mathrm{Li}} \cdot \lambda_{\mathrm{Li}}) \cdot I_{\mathrm{F}}}{(\sigma_{\mathrm{F}} \cdot \lambda_{\mathrm{F}})} \qquad (2)$$

From these calculations, we estimate that F^- can act as a counter-anion to roughly 70% of the total Li 1s intensity and LiTFSI to roughly 10%.

Supplementary References

1 Maibach, J. *et al.* A high pressure x-ray photoelectron spectroscopy experimental method for characterization of solid-liquid interfaces demonstrated with a Li-ion battery system. *Rev. Sci. Instrum.* **86**, 044101 (2015).

2 Goscinski, O., Müller, J., Poulain, E. & Siegbahn, H. Fluorine core esca linewidths in CH_3F and CF_4. *Chem. Phys. Lett.* **55**, 407-412, (1978).

3 Yeh, J. J. & Lindau, I. Atomic subshell photoionization cross sections and asymmetry parameters: $1 \leqslant Z \leqslant 103$. *Atomic Data and Nuclear Data Tables* **32**, 1-155, (1985).

4 Bhatt, M. D. & O'Dwyer, C. The Role of Carbonate and Sulfite Additives in Propylene Carbonate-Based Electrolytes on the Formation of SEI Layers at Graphitic Li-Ion Battery Anodes. *J. Electrochem. Soc.* **161**, A1415-A1421, (2014).
